# Health correlates and health-related outcomes associated with smartphone-related addictive behaviors in older adults: a scoping review

**DOI:** 10.3389/fpubh.2026.1879219

**Published:** 2026-07-31

**Authors:** Nan Jiang, Jie Yao, Yuchen Wu

**Affiliations:** School of Nursing, Shaanxi University of Chinese Medicine, Xianyang, Shaanxi, China

**Keywords:** nursing care, older adults, problematic smartphone use, scoping review, short-video overuse, smartphone-related addictive behaviors

## Abstract

**Background:**

Smartphones support communication, access to services, and social participation in later life, but a small and growing evidence base addresses patterns of use characterised by loss of control and functional interference among older adults.

**Objective:**

To map the concepts, assessment tools, health correlates, and nursing-care implications reported for smartphone-related addictive behaviors in adults aged 60 years or older.

**Methods:**

A scoping review was conducted in accordance with Joanna Briggs Institute guidance and reported with the PRISMA extension for Scoping Reviews. PubMed, Embase, CINAHL, Web of Science, Cochrane Library, CNKI, Wanfang, VIP, and SinoMed were searched from inception to 3 June 2026, and the reference lists of included studies were screened. Eligible original studies involved adults aged 60 years or older and examined smartphone addiction, mobile phone dependence, problematic smartphone or mobile phone use, smartphone overdependence, or short-video-related addictive behaviors primarily undertaken through smartphones. Evidence was synthesized descriptively and interpreted according to study design, measurement applicability, and directness to nursing care.

**Results:**

Twenty-two studies were included: 18 cross-sectional studies, 2 longitudinal studies, 1 randomized controlled trial, and 1 qualitative study. Concepts and measures were heterogeneous. Evidence was most concentrated for associations with poorer sleep quality, depressive symptoms, anxiety, and loneliness. Evidence concerning physical activity, instrumental activities of daily living, posture, visual fatigue, and social participation was more limited. Cognitive evidence was insufficient to support claims of decline. Direct intervention evidence was restricted to one small randomized trial reporting improvements in posture and neck function after home-based corrective exercise. The evidence base was geographically concentrated in China and other Asian settings, and age-stratified findings for the oldest-old were rarely reported.

**Conclusion:**

Current evidence supports cautious clinical inquiry, assessment of problematic use, and balanced health education rather than formal screening or definitive intervention recommendations. Future work should validate later-life-specific definitions and assessment thresholds, use prospective multicenter designs, report age-subgroup findings, and test nursing-led interventions that measure both behavioral and health-related outcomes.

## 1 Introduction

Population aging and digitalization are occurring simultaneously. The global population aged 65 years or older is projected to increase from 761 million in 2021 to 1.6 billion by 2050 (1). Smartphones have become embedded in later-life communication, payment, information seeking, entertainment, and health-related activities. In China, a Beijing survey of adults aged 60 years or older reported that 82.7% used smartphones for social communication and 38.2% used audiovisual entertainment functions (2). A national report on short-video use among older adults further showed that most users watched for less than 2 h per day, whereas only a small proportion reported very prolonged daily viewing (3). These findings emphasize that smartphone engagement is common in later life and that frequent use alone should not be labelled as addiction.

Smartphone-related addictive behaviors are characterised by difficulty controlling use and by adverse interference with daily routines, psychological well-being, or social functioning (4). In older adults, problematic use may be shaped by retirement-related changes in time use, reduced face-to-face companionship, social participation, physical limitations, and the availability of mobile entertainment. However, smartphones can also maintain social relationships and support everyday independence. This dual role requires a careful distinction between purposeful digital engagement and use patterns accompanied by loss of control or functional consequences.

The initial literature on smartphone addiction has largely focused on adolescents and younger adults. More recent older-adult studies have examined sleep, depressive symptoms, loneliness, physical activity, instrumental activities of daily living, posture, and short-video-related behaviors (5–8). Yet the evidence is difficult to interpret because studies use different concepts and instruments, many scales were developed in younger samples, most available studies are cross-sectional, and direct intervention evidence is sparse. These limitations are particularly important for nursing practice, where recommendations should not exceed the strength of the evidence.

The purpose of this scoping review was therefore to map the concepts, measurement instruments, health correlates, health-related outcomes, and nursing-care implications of smartphone-related addictive behaviors in older adults, while explicitly identifying limitations in causality, geographic generalizability, age-subgroup reporting, measurement thresholds, and intervention evidence.

## 2 Methods

### 2.1 Review design and rationale

A scoping review design was selected because the research question concerns a broad and heterogeneous body of evidence: different behavioral labels, multiple measurement tools, varied health outcomes, and very limited intervention research. The purpose was to map the available evidence and clarify gaps relevant to nursing care, rather than to calculate a pooled effect or determine comparative intervention efficacy. This approach offers greater methodological transparency than a narrative synthesis alone and is appropriate where the concepts, measures, and study designs have not yet converged. The review followed Joanna Briggs Institute guidance for scoping reviews and was reported in accordance with PRISMA-ScR (9–11).

### 2.2 Review questions

- Which concepts and assessment tools have been used to identify smartphone-related addictive behaviors in older adults?
- Which health problems and related factors have been reported in association with these behaviors?
- What direct nursing or health-management intervention evidence is available, and what nursing-care implications can be stated without overstating causality or effectiveness?

### 2.3 Eligibility criteria

Eligibility criteria were developed using the Population-Concept-Context framework. For this review, the umbrella term smartphone-related addictive behaviors was used to denote use patterns involving impaired control, salience or preoccupation, continued use despite negative consequences, and interference with daily functioning. Smartphone addiction, mobile phone addiction/dependence, problematic smartphone or mobile phone use, and smartphone overdependence were treated as device-level constructs. Social-media addiction and short-video addiction or overuse were treated as application-specific constructs and were eligible only when the behavior was primarily undertaken through smartphones or mobile devices and was assessed as uncontrolled, dependent, addictive, or functionally problematic. These application-specific studies were synthesized separately from general smartphone-use studies because they do not measure an identical construct.

Studies using the Internet Addiction Test or the Bergen Social Media Addiction Scale were retained only when the source article explicitly linked the problematic behavior to smartphone or mobile-device use among older adults. These instruments were not interpreted as smartphone-specific measures. Instead, they were classified as indirect or context-specific evidence and were used to inform the conceptual map without contributing to claims about the prevalence or severity of smartphone addiction itself (see Table 1).

**Table 1:** Eligibility criteria based on the population-concept-context framework.

PCC element	Inclusion criteria	Exclusion criteria	Rationale
Population	Adults aged ≥ 60 years, or studies reporting separable older-adult data	No separable older-adult data	Addresses later-life applicability and nursing relevance
Concept	Smartphone addiction; mobile phone addiction/dependence; problematic smartphone/mobile phone use; smartphone overdependence; smartphone-based short-video addiction/overuse	General internet use or digital literacy without problematic/addictive behavior outcomes	Maps related but non-identical behavioral constructs
Context	Community, home, residential care, outpatient, or other everyday older-adult care contexts	No relevant older-adult context	Supports public health and nursing interpretation
Evidence type	Original quantitative, qualitative, longitudinal, or intervention studies; Chinese or English full text	Reviews, editorials, case reports, duplicate publications, unavailable full text, non-Chinese/non-English texts	Enables mapping of primary evidence while documenting language limitation

### 2.4 Information sources and search strategy

Nine electronic databases were searched from inception to 3 June 2026: PubMed, Embase, CINAHL, Web of Science Core Collection, Cochrane Library, China National Knowledge Infrastructure (CNKI), Wanfang Data, VIP Database, and the Chinese Biomedical Literature Service System (SinoMed). Reference lists of included studies were also checked to identify eligible records. Search concepts combined terms for older adults with terms for smartphone or mobile phone addiction, dependence, overuse, problematic use, overdependence, and smartphone-based short-video addiction or overuse. Chinese and English publications were eligible. The full reproducible search strategies for each database are supplied in Supplementary Table S1.

### 2.5 Study selection and data charting

Records were imported into EndNote X9 for deduplication. Two reviewers independently screened titles and abstracts and then assessed potentially eligible full texts. Disagreements were discussed and referred to a third reviewer where consensus could not be reached. Full-text exclusion reasons were recorded in predefined categories, including no separable older-adult data, no smartphone- or mobile-device-related addictive/problematic behavior construct, general internet use or digital literacy without an addictive/problematic behavior outcome, application-specific behavior not primarily undertaken through smartphones, non-original publication type, unavailable full text, language outside Chinese or English, and duplicate or overlapping publication.

A standardized charting form captured author, year, country or study setting, recruitment setting, design, sample size, participant age and sex distribution where reported, behavioral concept, assessment instrument, measurement threshold or classification rule where reported, health outcomes, adjusted variables or analytic model, intervention content, main findings, and study limitations.

### 2.6 Critical appraisal approach and synthesis

In keeping with the mapping purpose of a scoping review, no study was excluded solely because of methodological limitations and no pooled risk-of-bias score was calculated. To strengthen critical interpretation, the synthesis considered: (1) study design and its capacity to support temporal or causal inference; (2) directness of the population to adults aged 60 years or older; (3) applicability of the instrument and threshold to later-life circumstances; (4) directness of each outcome to the claimed health problem; and (5) whether an intervention study assessed addictive behavior itself or only a related functional outcome. Findings from cross-sectional studies are therefore described as associations, mechanistic explanations are presented as hypotheses, inconsistent results are stated explicitly, and intervention implications are limited to outcomes directly evaluated. The interpretation framework is detailed in Supplementary Table S2.

## 3 Results

### 3.1 Study selection

**Figure 1:**
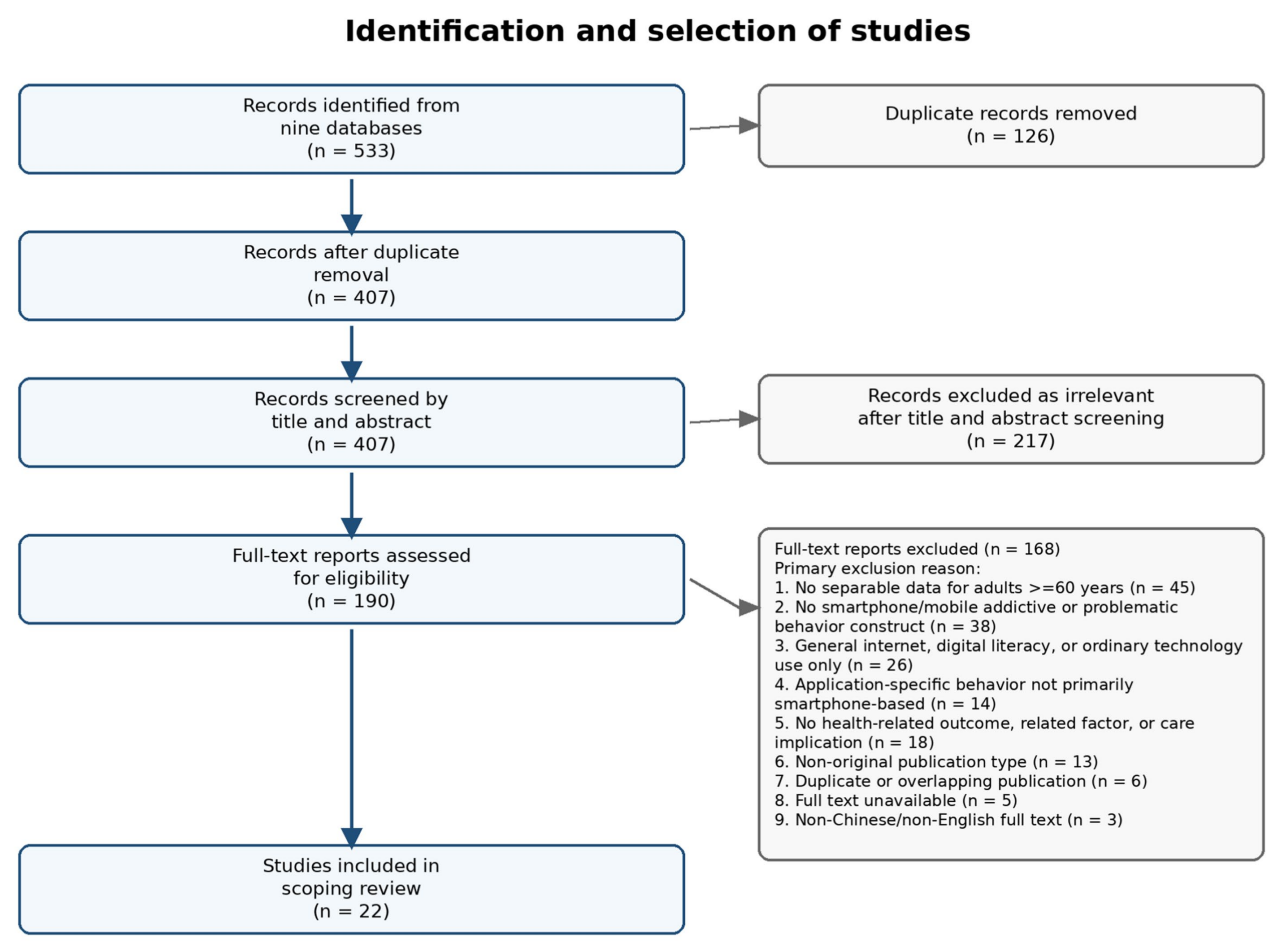
PRISMA-ScR flow diagram of study selection.

The search identified 533 records. After removal of 126 duplicates, 407 records underwent title and abstract screening. A total of 217 records were excluded as irrelevant at this stage, and 190 full-text reports were assessed for eligibility. After 168 full-text exclusions, 22 studies were included in the final synthesis. Using the primary reason for exclusion, the full-text exclusions were categorized as follows: no separable data for adults aged 60 years or older (*n* = 45); no smartphone- or mobile-device-related addictive/problematic behavior construct (*n* = 38); general internet use, digital literacy, or ordinary technology use without an addictive/problematic behavior outcome (*n* = 26); application-specific behavior not primarily undertaken through smartphones (*n* = 14); no relevant health-related outcome, related factor, or care implication reported (*n* = 18); non-original publication type, such as review, editorial, protocol, commentary, or case report (*n* = 13); duplicate or overlapping publication (*n* = 6); full text unavailable (*n* = 5); and non-Chinese/non-English full text (*n* = 3). These categories sum to 168 and were added to the PRISMA-ScR flow diagram. Figure 1 presents the study selection pathway.

### 3.2 Characteristics of included studies

Twenty-two studies published between 2019 and 2026 were included. Eighteen were cross-sectional studies, two were longitudinal studies, one was a randomized controlled trial, and one was a qualitative study. Evidence was geographically concentrated in China and other Asian settings; studies outside Asia were limited to Brazil and Türkiye. Nineteen studies examined smartphone addiction, mobile phone dependence, problematic mobile phone use, or smartphone overdependence, while three focused on short-video-related addictive behaviors in smartphone-use contexts. Table 2 provides expanded study-level information, including participant age, sex distribution, recruitment setting, measurement threshold or scoring approach, adjusted variables or analytic model, and main findings.

**Table 2:** Expanded characteristics of the included studies (*n* = 22).

No.	Study, region, and design	Sample and age	Sex distribution	Recruitment setting/Source	Concept, measure, and threshold	Adjusted variables/analytic model	Main findings
1	Bertocchi et al. (2022) (21) Brazil; Cross-sectional/population-based survey	668 community-dwelling older adults in total; 172 smartphone users analyzed for digital addiction Age: Older adults; exact mean/SD NR	NR	Population-based survey in a low-income Brazilian city; community-dwelling older adults	Digital addiction among smartphone users; Internet Addiction Test (IAT) Threshold/scoring: IAT score; smartphone-specific older-adult threshold NR	Structural equation modeling among smartphone users; exact covariate adjustment NR	Smartphone ownership was associated with younger age, higher income, higher MMSE, more social support and less dependence. Among smartphone users, digital addiction correlated with poorer sleep; hours of use were not correlated with health outcomes.
2	Chen et al. (2025) (31) China; Longitudinal, three-wave survey	828 older adults Age: ≥ 60 years; mean/SD NR	Male 382 (46.1%); female 446 (53.9%)	Senior universities in Jiangxi, Hunan, Guizhou and Chongqing; classroom-based paper survey, Apr 2024–Jan 2025	Mobile phone addiction tendency; Mobile Phone Addiction Tendency Scale (MPATS) Threshold/scoring: 16 items, 5-point Likert; higher score indicates greater tendency; no cutoff reported; used as continuous score	Repeated-measures ANOVA and cross-lagged panel model; gender/time effects examined	Positive exercise experience and mobile phone addiction tendency were negatively correlated. Positive exercise experience predicted lower later mobile phone addiction tendency, and mobile phone addiction tendency predicted lower later positive exercise experience from T1 to T2.
3	Chen et al. (2026) (35) China; Cross-sectional survey with ML and fsQCA	2,585 community-dwelling older adults Age: 60–69: 1,490 (57.6%); 70–79: 729 (28.2%); ≥ 80: 366 (14.2%)	Male 866 (33.5%); female 1,719 (66.5%)	87 social workstations/community centers in five districts of Guangzhou; on-site assisted survey, Oct-Nov 2024	Smartphone addiction; 17-item Mobile Phone Addiction Index (MPAI) Threshold/scoring: 5-point Likert; summed and standardized/used in models; no older-adult-specific cutoff reported	Machine learning and fsQCA; variables included gender, age, education, employment, self-rated health, income, marital status and daily smartphone use	Random forest showed strongest prediction of depression. Smartphone addiction and social participation were key predictors; high smartphone addiction was a central condition in fsQCA configurations associated with depression.
4	Demir et al. (2025) (7) Türkiye; Descriptive cross-sectional study	94 older adults; smartphone addiction group *n* = 45, non-addiction group *n* = 49 Age: Mean age 70.67 ± 4.24 years; inclusion age ≥ 65	Female 51.1%; male 48.9%	YADEM Sakarya Metropolitan Municipality Elderly Support and Coordination Center; face-to-face assessment, Mar-Jun 2024	Smartphone addiction; Smartphone Addiction Scale-Short Version (SAS-SV) Threshold/scoring: 10 items, 6-point Likert, total 10–60; cutoff 31 for women and 33 for men	Independent t test, chi-square and structural equation/path analysis; no conventional covariate-adjusted regression reported	The smartphone-addiction group had lower physical activity and IADL scores. Physical activity and IADL negatively predicted SAS-SV score; balance did not differ significantly.
5	Ding et al. (2024) (23) China; Cross-sectional community survey	376 valid respondents Age: Mean age approximately 69 years	Male 161 (42.8%); female 215 (57.2%)	Two communities in Wuhan, Hubei Province; offline face-to-face survey, Jan-Feb 2023	Problematic mobile phone use; Mobile Phone Problem Use Scale-10 (MPPUS-10) Threshold/scoring: 10 items, 10-point Likert, score 10–100; cutoff mentioned as 41; higher score indicates more problematic use	Linear regression and Hayes PROCESS Model 6; demographics and mobile phone use variables were included as controls	Problematic mobile phone use was associated with depressive symptoms through social support and attitudes toward aging. Female sex, living with spouse and later marriage age were linked to lower MPPU; older age and unemployment were linked to higher MPPU.
6	Hao and Wu (2026) (33) China; Cross-sectional/latent profile analysis	560 older adults Age: NR	NR	Urban sub-health older adults; detailed recruitment setting NR	Mobile phone dependence; modified MPAI Threshold/scoring: Modified MPAI; threshold/scoring details NR	Latent profile analysis; self-control and Tai Chi exercise considered according to title/manuscript	Manuscript summary indicates profiles involving mobile phone dependence, self-control and Tai Chi exercise; full model findings need verification from original article.
7	Jeong and Bae (2022) (36) South Korea; Cross-sectional secondary analysis	3,121 older smartphone users Age: Mean age 63.83 ± 2.88 years; age 60–69	Male 1,627 (52.1%); female 1,494 (47.9%)	2019 National Information Society Agency survey; stratified household survey across 17 regions; trained interviewer visits	Smartphone addiction/overdependence; Korean S-scale Threshold/scoring: 10 items, 4-point scale; high risk > = 28, potential risk 24–27; addiction-risk group defined as high + potential risk	Hierarchical multiple regression: sociodemographics and usage time, smartphone use types, and digital literacy entered sequentially	Addiction-risk prevalence was 14.6%. Longer usage time, entertainment/life-service use and digital literacy were positively associated with smartphone addiction; information-seeking and communication-seeking use were negatively associated.
8	Karas et al. (2023) (22) Türkiye; Cross-sectional study	392 older adults Age: NR	NR	Türkiye; detailed recruitment setting NR	Addictive smartphone use; Smartphone Addiction Scale (SAS) Threshold/scoring: SAS threshold/scoring details NR	NR	Manuscript summary indicates relationships with depression, anxiety and sleep quality; numerical findings need verification from original article.
9	Kong et al. (2026) (37) South Korea; Cross-sectional secondary analysis/machine learning	4,338 older adults aged > = 60 Age: 60–64: 2,246 (51.8%); ≥ 65: 2,092 (48.2%)	Male 2,141 (49.4%); female 2,197 (50.6%)	2023 National Information Society Agency Smartphone Overdependence Survey; nationwide household survey	Smartphone overdependence; Korean S-scale Threshold/scoring: 10 items, 4-point scale; high-risk > = 28, potential risk 24–27; study coded overdependence/at-risk as score > = 24	Machine-learning models using 80 explanatory variables; feature importance rather than conventional adjusted regression	XGBoost performed best for predicting overdependence. Important predictors included demographics, smartphone-use time composition, overdependence awareness and content-use variables.
10	Lai et al. (2025) (6) China; Cross-sectional survey with mediation analysis	200 older adults Age: Mean age 64.72 ± 3.21 years; inclusion age 60–70	Male 126 (63.0%); female 74 (37.0%)	Foshan area: three medical institutions, three older-adult care institutions and community households; Aug 2022-Aug 2023	Smartphone addiction; SAS-SV Threshold/scoring: SAS-SV 10 items, 6-point Likert, total 10–60; score > = 21 used to indicate higher smartphone addiction	Linear regression and PROCESS mediation analysis; age and gender controlled in mediation model	Smartphone addiction was associated with poorer sleep quality. Depression and loneliness partially mediated the association between smartphone addiction and sleep quality.
11	Shete et al. (2020) (34) India; Cross-sectional study	100 older adults Age: Geriatric population; exact age distribution NR	NR	India; detailed recruitment setting NR	Smartphone addiction; Mobile Phone Addiction Scale (MPAS) Threshold/scoring: MPAS threshold/scoring details NR	NR	Manuscript summary indicates assessment of smartphone addiction and reaction time; detailed findings need verification from original article.
12	Tian and Wang (2023) (5) China; Cross-sectional survey with mediation analysis	459 valid respondents Age: Adults >60 years; mean/SD NR	Male 43.1%; female 56.9%	Communities in Huai’an and Nanjing, Jiangsu Province; community recruitment with trained data collectors	Mobile phone addiction; SAS-SV Threshold/scoring: SAS-SV 10 items, 6-point Likert; higher score indicates higher addiction; cutoff NR	Correlation and PROCESS mediation/bootstrapping; no covariate adjustment clearly reported	Mobile phone addiction was associated with poorer sleep quality. Depression and loneliness partially mediated the association, with direct and indirect effects reported.
13	Vannajak et al. (2025) (8) Thailand; Randomized controlled trial	60 older adults with smartphone addiction; CON/HARD/SOFT groups *n* = 20 each Age: Mean age 66.5 ± 4.5 years	NR	Thailand; home-based intervention; detailed recruitment setting NR	Smartphone addiction; SAS-SV according to manuscript table Threshold/scoring: Threshold for identifying smartphone addiction NR	Mixed-model ANOVA comparing group, time and interaction effects	Six-week home-based corrective exercise improved forward-head posture, rounded shoulder posture and deep cervical flexor function. Balance, body composition and quality of life did not change significantly; addictive behavior itself was not shown to improve.
14	Chen and Dai (2023) (24) China; Cross-sectional moderated mediation study	236 older adults Age: NR	NR	China; recruitment setting NR	Mobile phone dependence; MPAI Threshold/scoring: MPAI threshold/scoring details NR	Moderated mediation model; sex moderation indicated by manuscript topic	Manuscript summary indicates relationships among social support, loneliness, mobile phone dependence and sex moderation; detailed estimates need verification from original article.
15	Huang et al. (2024) (32) China; Longitudinal cross-lagged study	859 older adults Age: NR	NR	China; recruitment setting NR	Mobile phone addiction; MPATS Threshold/scoring: MPATS threshold/scoring details NR	Cross-lagged model according to manuscript topic	Manuscript summary indicates a cross-lagged relationship between positive exercise experience and mobile phone addiction; detailed findings need verification from original article.
16	Tang et al. (2019) (27) China; Cross-sectional study	213 middle-aged/older adults or older-adult subgroup according to manuscript Age: NR	NR	Zunyi City according to manuscript reference; detailed recruitment setting NR	Mobile phone addiction; MPAI Threshold/scoring: MPAI threshold/scoring details NR	NR	Manuscript discussion indicates no statistically significant association between mobile phone addiction and loneliness in older-adult subgroup; detailed estimates need verification from original article.
17	Wang et al. (2022) (38) China; Cross-sectional study	205 community-dwelling older adults Age: NR	NR	Community-dwelling older adults according to manuscript reference; detailed setting NR	Mobile phone dependence; MPAI Threshold/scoring: MPAI threshold/scoring details NR	NR	Manuscript summary indicates associated factors including age, employment, satisfaction and child care; detailed estimates need verification from original article.
18	Zhang et al. (2024) (26) China; Qualitative interview study	27 older adults and 8 relatives Age: NR	NR	Interview study; detailed recruitment setting NR	Smartphone dependence; qualitative interviews Threshold/scoring: Not applicable; qualitative study	Qualitative thematic/generative mechanism analysis	Manuscript summary indicates generative mechanisms involving social contact, family interaction and sleep; detailed themes need verification from original article.
19	Zhang et al. (2019) (25) China; Cross-sectional study	206 older patients Age: NR	NR	Older patients according to manuscript reference; detailed setting NR	Mobile phone dependence; MPAI Threshold/scoring: MPAI threshold/scoring details NR	NR	Manuscript summary indicates relationship between mobile phone dependence and loneliness; detailed estimates need verification from original article.
20	Cui et al. (2026) (28) China; Cross-sectional study; short-video-specific evidence	384 older adults Age: NR	NR	China; recruitment setting NR	Short-video/social-media addiction; Bergen Social Media Addiction Scale (BSMAS) Threshold/scoring: BSMAS is not smartphone-specific; threshold/scoring details for older adults NR	NR	Manuscript summary indicates associations among social support, connectedness and satisfaction in short-video addiction context; detailed estimates need verification from original article.
21	Dong et al. (2025) (29) China; Cross-sectional study; short-video-specific evidence	872 rural older adults Age: NR	NR	Rural older adults; detailed recruitment setting NR	Short-video addiction tendency; Short Video Addiction Scale (SVAS) Threshold/scoring: SVAS threshold/scoring details NR	NR	Manuscript summary indicates associations with depression, visual fatigue and sleep; detailed estimates need verification from original article.
22	Li et al. (2025) (30) China; Cross-sectional mediation study; short-video-specific evidence	314 older adults Age: NR	NR	China; recruitment setting NR	Short-video overuse; adapted short-video scale Threshold/scoring: Adapted scale threshold/scoring details NR	Chain mediation model indicated by manuscript topic	Manuscript summary indicates social isolation influences short-video overuse through loneliness and online social support; detailed estimates need verification from original article.

The table reports participant age, sex distribution, recruitment setting, measurement threshold or scoring approach, adjusted variables or analytic model, and main findings where available. NR, not reported in the accessible article information or not extractable from the available full text/abstract; IADL, instrumental activities of daily living; IAT, Internet Addiction Test; MPAI, Mobile Phone Addiction Index; MPATS, Mobile Phone Addiction Tendency Scale; MPPUS-10, Mobile Phone Problem Use Scale-10; SAS, Smartphone Addiction Scale; SAS-SV, Smartphone Addiction Scale-Short Version; SVAS, Short Video Addiction Scale; RCT, randomized controlled trial; ML, machine learning; fsQCA, fuzzy-set qualitative comparative analysis.

### 3.3 Concepts and measurement instruments

The included studies used several overlapping but non-identical labels. Smartphone addiction and mobile phone dependence generally refer to device-level patterns marked by impaired control, salience, tolerance-like escalation, withdrawal-like discomfort, and interference with daily life. Problematic smartphone or mobile phone use is a broader and less diagnostic term that emphasizes adverse consequences or maladaptive use without necessarily assuming an addiction model. Smartphone overdependence, particularly in Korean surveillance research, similarly captures excessive reliance and impaired control but is embedded in a public-health risk framework rather than a clinical diagnosis.

Social media addiction and short-video addiction or overuse are application-specific constructs. They may occur through smartphones, but their content environment, reinforcement mechanisms, and social functions differ from general smartphone use. In this review, these studies were therefore used as supplementary evidence for smartphone-mediated problematic behaviors in later life and were not combined with device-level smartphone-addiction studies for prevalence, severity, or causal interpretation.

Measures included the Internet Addiction Test (IAT), Mobile Phone Addiction Index (MPAI), Mobile Phone Addiction Tendency Scale (MPATS), Mobile Phone Problem Use Scale-10 (MPPUS-10), Smartphone Addiction Scale (SAS), SAS-Short Version (SAS-SV), the Korean smartphone overdependence S-scale, the Bergen Social Media Addiction Scale (BSMAS), and short-video-related instruments (12–20). The MPAI, MPATS, MPPUS-10, SAS, SAS-SV, and S-scale were considered closer to device-level smartphone or mobile-phone constructs. By contrast, the IAT and BSMAS were considered indirect or application-specific measures: they were included only when the original study context explicitly linked the assessed behavior to smartphone or mobile-device use among older adults.

Instrument suitability for older adults remains uncertain. Several instruments were originally developed among adolescents, students, or general internet users, and some items refer to school, work, or role disruption that may be less salient after retirement. In later life, functional interference may instead be reflected by disrupted sleep, reduced outdoor activity, interference with medication or health-management routines, neglect of household responsibilities, visual or musculoskeletal discomfort, or withdrawal from face-to-face interaction. Prolonged but purposeful smartphone use for communication, payment, health management, or social participation should not be interpreted as addictive behavior unless accompanied by impaired control or functional consequences. No uniform, validated later-life-specific severity threshold emerged from the included evidence.

### 3.4 Health correlates and related factors

#### 3.4.1 Sleep and psychological health

Sleep quality, depressive symptoms, anxiety, and loneliness were the most frequently examined health correlates. Cross-sectional studies in older adults reported that higher levels of smartphone-related addictive behavior were associated with poorer sleep quality and higher depression, anxiety, or loneliness scores (5, 6, 21, 22). Tian and Wang and Lai et al. reported statistical mediation models involving depression and loneliness in the association between phone addiction and sleep quality (5, 6). These models identify plausible relational pathways but do not establish temporal order or causation.

Other studies identified associations between problematic mobile phone use and depressive symptoms, social support, and attitudes to aging, as well as associations between mobile phone dependence, social support, and loneliness (23–25). A qualitative study described emotional needs, changes in social connection, and intergenerational interaction as possible contexts for smartphone dependence (26). Findings were not fully consistent: Tang et al. did not detect a statistically significant association between mobile phone addiction and loneliness in their older-adult subgroup (27).

#### 3.4.2 Application-specific short-video evidence

Application-specific short-video evidence is reported separately here because short-video addiction or overuse reflects a content-specific and platform-mediated behavior rather than general smartphone addiction. Studies of short-video-related addictive behaviors among older adults reported associations with depressive symptoms, visual fatigue, reduced sleep efficiency, subjective social isolation, loneliness, online social support, and life satisfaction (28–30). These findings support attention to loss of control and functional impact in short-video settings, but they do not justify treating ordinary short-video entertainment, or prolonged viewing alone, as addiction.

#### 3.4.3 Physical activity, daily function, posture, and cognition

Evidence for physical and functional outcomes was less extensive. In a Turkish cross-sectional study, older adults classified in the smartphone-addiction group had lower physical activity and instrumental activities of daily living scores than those outside that group, whereas balance did not differ significantly (7). Two longitudinal studies reported reciprocal or prospective associations between more positive exercise experiences and lower subsequent mobile phone addiction tendencies (31, 32). A further cross-sectional study identified associations between Tai Chi exercise, self-control, and mobile phone-dependence profiles (33). These findings are compatible with an interaction between mobile phone behavior and active lifestyles, but they do not show that reducing smartphone use alone will improve physical function.

Direct intervention evidence was restricted to one randomized controlled trial of 60 older adults identified as having smartphone addiction. Home-based corrective exercise improved forward-head posture, rounded shoulder posture, and deep cervical flexor function (8). The trial provides preliminary direct evidence for managing postural outcomes in a selected population, but it did not establish improvement in addictive behavior, sleep, mood, or social participation. With respect to cognition, a single study reported an association with simple reaction time (34); this evidence is insufficient to support a conclusion that smartphone-related addictive behaviors cause cognitive decline in older adults.

#### 3.4.4 Social support, use patterns, and digital context

Social support, life satisfaction, living arrangements, care from adult children, social participation, and use preferences were repeatedly identified as correlates of smartphone-related addictive behaviors (23–26, 35–38). These findings suggest that problematic use in later life should not be interpreted solely as deficient self-control; it may also occur in the context of unmet emotional needs, reduced offline interaction, or restricted participation opportunities. However, reverse causation is plausible: loneliness, depression, limited mobility, or low participation may increase reliance on smartphones rather than result from it.

### 3.5 Evidence relevant to nursing care

Direct nursing-intervention evidence remains limited. The single randomized controlled trial addressed home-based corrective exercise for posture and neck function, rather than the addictive behavior construct itself (8). Therefore, the evidence supports practical considerations and assessment priorities more strongly than it supports established nursing intervention protocols.

For clinical inquiry and assessment, nurses and community health professionals may ask about difficulty stopping use, perceived loss of control, bedtime use patterns, interruption of sleep or daily routines, mood concerns, loneliness, social support, physical activity, visual fatigue, and postural discomfort. Because validated older-adult thresholds are not yet established, these questions should not be presented as formal screening or diagnostic classification. For education, it is reasonable to discuss balanced digital routines, pauses during prolonged viewing, ergonomics, physical activity, and the distinction between beneficial use and functionally disruptive use. For individuals presenting with depressive symptoms, anxiety, marked loneliness, impaired function, or persistent sleep complaints, assessment and referral should follow established clinical pathways; current evidence does not show that smartphone-use guidance alone resolves these outcomes.

For short-video behaviors, assessment should remain separate from general smartphone-use assessment and should emphasize loss of control, distress, and interference with offline activities rather than viewing time alone. Family or community participation may be considered when poor offline support is identified, while recognizing that the effectiveness of these approaches for reducing smartphone-related addictive behaviors has not yet been tested adequately.

## 4 Discussion

### 4.1 Principal findings and strength of evidence

This review maps an emerging but methodologically uneven evidence base concerning health correlates and health-related outcomes of smartphone-related addictive behaviors in older adults. Studies were most numerous for sleep and psychological health, with repeated reports of associations between device-level problematic use and poorer sleep, depressive symptoms, anxiety, or loneliness. Application-specific short-video studies reported related but distinct associations and were interpreted separately. The evidence for physical activity, daily function, posture, visual fatigue, and social participation was less extensive, and evidence for cognition was sparse. Most importantly for clinical interpretation, 18 of the 22 included studies were cross-sectional. The available literature therefore documents associations and potential care needs more clearly than it identifies causation or effective treatment pathways.

Evidence for nursing intervention is especially limited. One small randomized controlled trial provides direct support for home-based corrective exercise in improving particular postural and neck-function outcomes, but there is insufficient evidence to conclude that nursing-led sleep education, psychological support, family intervention, community participation programs, or digital health education reduce smartphone-related addictive behaviors themselves. These approaches should currently be framed as care implications and candidate intervention components requiring evaluation.

### 4.2 Evidence interpretation by outcome domain

### 4.3 Measurement validity and unclear severity thresholds

A major limitation is the lack of a validated, later-life-specific definition and threshold. Instruments applied in the included studies were originally developed across different populations and constructs, including internet addiction, student mobile phone addiction, smartphone addiction, smartphone overdependence, social-media addiction, and short-video addiction. The IAT and BSMAS require particular caution because they do not specifically assess smartphone-related addictive behaviors unless the source study clearly links the behavior to smartphone or mobile-device use. Items concerning school or employment disruption may misclassify retired adults, while prolonged purposeful use for communication, payment, or health management should not be interpreted as addiction without evidence of loss of control or impaired function. Future tool development should incorporate daily-life interference relevant to older adults and should examine reliability, construct validity, criterion validity, and meaningful risk thresholds in different age groups (see Table 3).

**Table 3:** Strength and interpretive limits of evidence across outcome domains.

Outcome domain	Nature of current evidence	Interpretation supported by evidence	Key gap
Sleep quality and psychological health	Several older-adult studies; predominantly cross-sectional; inconsistent loneliness finding	Associations are relatively consistently reported; direction of effect remains uncertain	Prospective cohorts and intervention trials
Physical activity and IADL	Few cross-sectional and longitudinal studies	Initial associations with activity patterns and functional status	Temporal direction and modifiable pathways
Posture and neck function	One small RCT plus observational evidence	Preliminary support for corrective exercise on specific postural outcomes	Effects on addictive behavior and broader health outcomes
Cognition	Very limited evidence, including reaction time	No conclusion about cognitive decline	Age-stratified longitudinal cognitive studies
Nursing interventions	Minimal direct evaluation	Use as assessment and research priorities rather than validated protocols	Multicomponent nursing-led trials

### 4.4 Causality, reverse causation, and evidence gaps

The predominance of cross-sectional designs substantially limits causal interpretation. Poor sleep may encourage nighttime smartphone use; depression or loneliness may increase reliance on mobile interaction or short-video content; reduced mobility or social participation may lead to greater sedentary screen time. Smartphone-related addictive behavior may also aggravate these problems. The evidence is therefore compatible with reciprocal pathways, rather than a unidirectional model in which smartphone use alone produces health problems. Proposed mechanisms, such as disrupted sleep routines or displacement of face-to-face interaction, remain hypotheses unless tested prospectively or experimentally in older adults.

Several substantive gaps require attention. First, the evidence base is geographically narrow: studies were concentrated in China and other Asian contexts, with limited representation from Latin America and no substantial included evidence from North America, Africa, or most of Europe. Cultural norms of family support, preferred platforms, digital literacy, and community services may influence both behavior and health correlations. Second, restricting eligibility to Chinese- and English-language publications creates potential language bias and may limit access for international readers to several Chinese reports. Third, most studies treated older adults as one group and did not compare adults aged 60–69 years, 70–79 years, and 80 years or older. Such subgroups may differ in device competence, physical vulnerability, sensory function, and social context. Fourth, prospective cohort studies linking behavior to later health outcomes are scarce, leaving the temporal sequence uncertain.

### 4.5 Implications for nursing care and future research

At present, nursing care should focus on recognition, clinical inquiry, individualized support, and appropriate referral rather than formal screening or standardized treatment protocols. Assessment should distinguish purposeful, beneficial smartphone engagement from use characterised by impaired control and disruption of sleep, activity, health management, or social life. Nursing education can address practical sleep routines, scheduled breaks, ergonomics, physical activity, and maintaining offline social contact, while avoiding the assumption that high screen time alone identifies addiction. These points should be framed as practical considerations and preliminary recommendations derived mainly from observational evidence, not as interventions with established effectiveness.

Research priorities include: development and validation of assessment tools and thresholds that reflect later-life contexts; multicenter prospective cohorts with age-subgroup reporting; studies across diverse geographic and cultural settings; and nursing-led intervention trials that measure both behavior-related outcomes and health outcomes such as sleep, mood, physical function, and social participation. Intervention studies should state whether their primary goal is reducing uncontrolled use, improving a related health problem, or both.

### 4.6 Strengths and limitations of this review

This review strengthens transparency by applying a scoping-review framework, searching nine Chinese and international databases, explicitly separating general smartphone-related constructs from application-specific short-video behaviors, and integrating design-sensitive interpretation throughout the synthesis. Several limitations remain. The included literature was dominated by cross-sectional designs and used heterogeneous measurement tools. The review included only Chinese- and English-language studies, and the evidence base was geographically concentrated. Formal quantitative pooling was not appropriate because of conceptual and methodological heterogeneity. In addition, because direct intervention evidence was minimal, the nursing implications described here should not be considered proven interventions for addictive behavior. The expanded extraction table also depends on the reporting quality of the original studies, and some participant or analytic details may not have been available in all source reports.

## 5 Conclusion

Smartphone-related addictive behaviors among older adults are an emerging public health and nursing concern, but current evidence remains at an early stage. Existing studies most consistently report cross-sectional associations with sleep and psychological health, while evidence concerning physical function, cognition, and intervention effectiveness is weaker. Application-specific short-video evidence suggests related concerns but should remain analytically distinct from general smartphone-addiction evidence. Tools and severity thresholds require later-life-specific validation, and current observational evidence cannot establish causal direction. Nursing practice can reasonably incorporate clinical inquiry and assessment of loss of control and functional interference, provide balanced digital-health and ergonomic education, and address coexisting sleep or psychosocial concerns through appropriate care pathways. Stronger prospective and intervention evidence is required before formal screening thresholds or specific nursing strategies can be regarded as validated approaches for smartphone-related addictive behaviors in older adults.

## Data Availability

No original datasets were generated for this review. Information extracted from published studies is presented in the manuscript and Supplementary Material. Full database search strategies are provided in Supplementary Table S1.
