# Supplementary material for: Health correlates and health-related outcomes associated with smartphone-related addictive behaviors in older adults: a scoping review
Source: Front Public Health. 2026 Jul 31;14:1879219. doi: 10.3389/fpubh.2026.1879219 (PMC13472799; doi:10.3389/fpubh.2026.1879219)
Supplement: Supplementary file 1 [file Supplementary_file_1.docx]

# Supplementary Table S1. Database search strategies used in this scoping review

Search strategies were adapted according to the indexing characteristics and search syntax of each database. Search concepts included older adults and smartphone-related addictive behaviors, including smartphone addiction, problematic smartphone/mobile phone use, mobile phone dependence, smartphone overdependence, and smartphone-based short-video-related addictive behaviors.

| Database | Search strategy concept |
| --- | --- |
| PubMed | Older adults AND smartphone-related addictive behaviors (smartphone addiction, problematic smartphone use, mobile phone dependence, mobile phone addiction, smartphone overdependence, short-video-related addictive behaviors) |
| Embase | Older adults AND smartphone/mobile phone addiction-related terms |
| CINAHL | Older adults AND problematic smartphone/mobile phone use-related terms |
| Web of Science Core Collection | Older adults AND smartphone addiction/problematic use-related terms |
| Cochrane Library | Older adults AND smartphone/mobile phone addiction-related terms |
| CNKI | 老年人 AND 手机成瘾、智能手机成瘾、手机依赖、手机过度使用、短视频相关成瘾行为 |
| Wanfang Data | 老年人 AND 手机成瘾、手机依赖、智能手机使用问题、短视频过度使用 |
| VIP Database | 老年人 AND 手机成瘾、手机依赖、智能手机成瘾 |
| SinoMed | 老年人 AND 手机成瘾、手机依赖、智能手机相关问题 |

# Supplementary Table S2. Interpretation framework used for evidence synthesis

The interpretation framework was applied to evaluate the relevance, directness, and strength of evidence identified in this scoping review.

| Domain | Interpretation principle |
| --- | --- |
| Study design | Cross-sectional studies were interpreted as reporting associations rather than causal relationships. Longitudinal and intervention studies were considered according to their ability to support temporal inference. |
| Population | Evidence was evaluated according to its directness to adults aged 60 years or older. |
| Measurement instruments | The applicability of assessment tools and thresholds to older adults was considered, especially whether instruments were originally developed in younger populations. |
| Health outcomes | Outcomes were interpreted according to their directness to reported health problems, including sleep, psychological health, physical function, cognition, and social participation. |
| Intervention evidence | Interventions were evaluated according to whether they directly targeted addictive behaviors or related functional outcomes. |
| Causal interpretation | Mechanistic explanations were considered hypotheses unless supported by appropriate longitudinal or experimental evidence. |
